# Molecular mechanisms of histone deacetylases and inhibitors in renal fibrosis progression

**DOI:** 10.3389/fmolb.2022.986405

**Published:** 2022-09-06

**Authors:** Jiayu Wang, Jiaxing Li, Xin Zhang, Min Zhang, Xiaopeng Hu, Hang Yin

**Affiliations:** ^1^ Department of Urology, Beijing Chao-Yang Hospital, Capital Medical University, Beijing, China; ^2^ Institute of Urology, Beijing Chao-Yang Hospital, Capital Medical University, Beijing, China; ^3^ Department of Research Ward, Beijing Chao-Yang Hospital, Capital Medical University, Beijing, China

**Keywords:** renal fibrosis, CKD, HDACs, HDAC inhibitors, TGF-β, BMP-7, smad, ECM

## Abstract

Renal fibrosis is a common progressive manifestation of chronic kidney disease. This phenomenon of self-repair in response to kidney damage seriously affects the normal filtration function of the kidney. Yet, there are no specific treatments for the condition, which marks fibrosis as an irreversible pathological sequela. As such, there is a pressing need to improve our understanding of how fibrosis develops at the cellular and molecular levels and explore specific targeted therapies for these pathogenic mechanisms. It is now generally accepted that renal fibrosis is a pathological transition mediated by extracellular matrix (ECM) deposition, abnormal activation of myofibroblasts, and epithelial-mesenchymal transition (EMT) of renal tubular epithelial cells under the regulation of TGF-β. Histone deacetylases (HDACs) appear to play an essential role in promoting renal fibrosis through non-histone epigenetic modifications. In this review, we summarize the mechanisms of renal fibrosis and the signaling pathways that might be involved in HDACs in renal fibrosis, and the specific mechanisms of action of various HDAC inhibitors (HDACi) in the anti-fibrotic process to elucidate HDACi as a novel therapeutic tool to slow down the progression of renal fibrosis.

## 1 Introduction

Chronic kidney disease (CKD) is a clinical syndrome characterized by persistent changes in kidney structure and function, which manifests as hypertension, edema, and oliguria, accompanied by abnormally elevated serum creatinine or blood urea nitrogen. The most common pathological manifestation of CKD is renal fibrosis (Romagnani et al., 2017). Renal fibrosis is chronic and progressive and seriously affects the glomerular filtration function. Data show that renal disease and interstitial fibrosis are present in approximately 50% of the over 70-year-old population and up to 10% of the world population. However, no specific anti-fibrotic drugs exist. Fortunately, recent advances have shed light on vital mechanisms of renal fibrosis development at the molecular level. Vorinostat (SAHA) is the first HDAC inhibitor approved by the FDA, mainly for the treatment of cutaneous T-cell lymphoma (Marks and Breslow, 2007). HDACi inhibits the proliferation and differentiation of tumors and can also promote apoptosis (Kim and Bae, 2011). Recent studies have shown that HDACi can also effectively inhibit the progression of renal fibrosis in various animal models such as ureteral ligation, diabetic nephropathy, ischemia-reperfusion, hypertensive nephropathy, and multiple sclerosis. As such, HDACs may be a potential target for antifibrotic effects, and HDACi may provide a clinically effective therapeutic to reverse renal fibrosis.

## 2 The mechanisms of renal fibrosis

**FIGURE 1:**
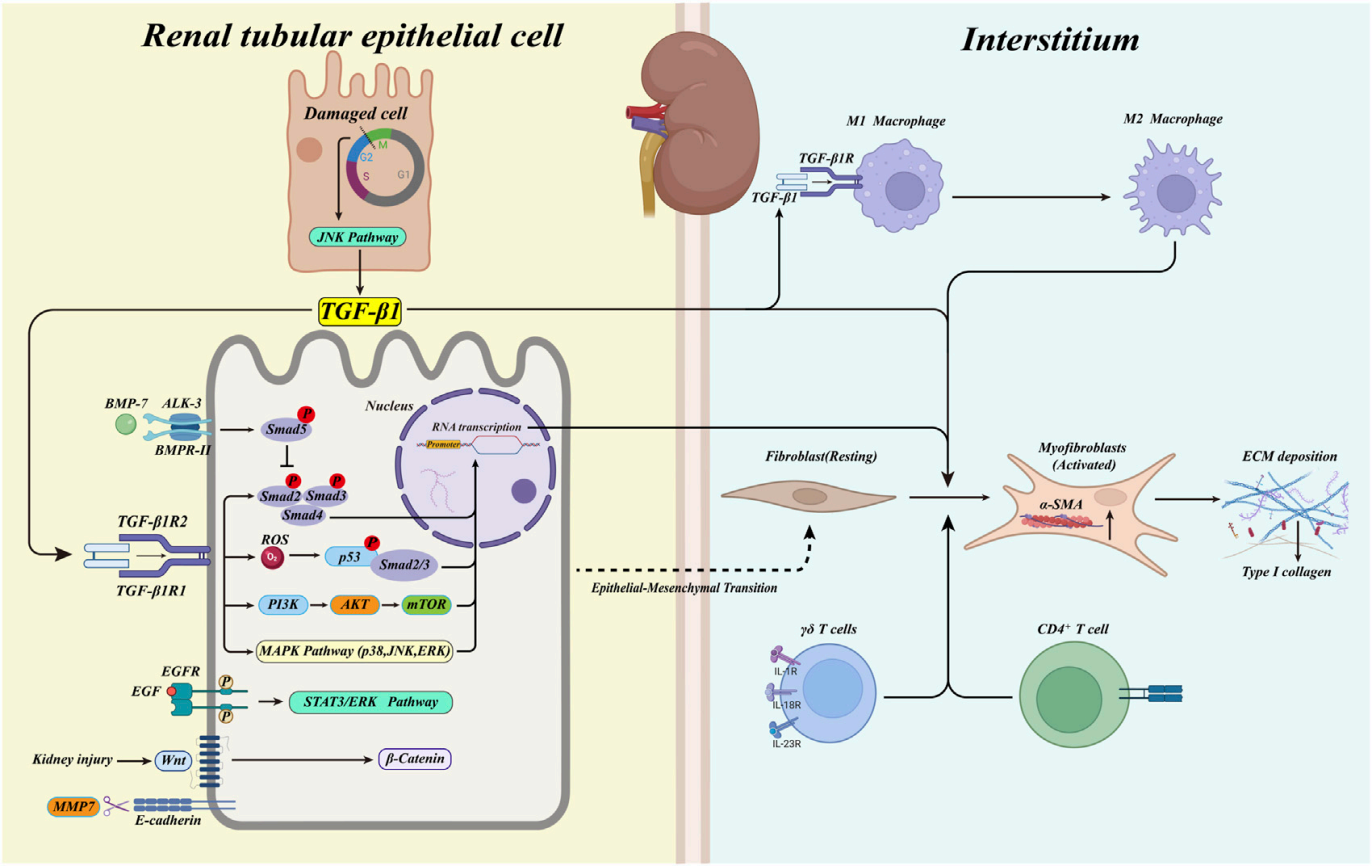
The activated TGF-β1/Smad pathway initiates the transcription of pro-fibrotic factors when the kidney damaged. At the same time, myofibroblasts in the renal interstitium are activated and produce large amounts of ECM. In response to TGF-β1, renal tubular epithelial cells undergo phenotypic conversion to fibroblasts (type 2 EMT). Immune cells infiltrate in the renal interstitium, triggering the deposition of ECM. Macrophages convert from type M1 to type M2 in response to TGF-β1, promoting fibrosis. BMP-7 activates the phosphorylation of Smad5, which plays a protective role against the TGF-β1/Smad pathway. Blocked G2/M phase of cell cycle in damaged tubular epithelial cells activates the JNK pathway and promotes the production of TGF-β1. Meanwhile, MAPK kinases activated by TGF-β1, p53, EGF, and Wnt/β-catenin pathways are also involved in the renal fibrosis process.

Renal fibrosis mainly stems from abnormal activation of myofibroblasts and their secretion of large amounts of ECM, such as α-smooth muscle actin (α-SMA), fibronectin (FN), and large deposits of collagen type I (collagen I), causing abnormal expansion of the renal tubular mesenchyme (Figure 1) (Humphreys, 2018). Tracking and localization assays using a genetic mouse model demonstrated the origin of mesenchymal myofibroblasts in the renal fibrosis model; half originated from the proliferative differentiation of resident fibroblasts with a contractile myofibroblast phenotype (Broekema et al., 2007; Meran and Steadman, 2011; LeBleu et al., 2013).

It is now generally accepted that the initial histological hallmark of renal fibrosis progression is the accumulation of myofibroblasts in the renal interstitium, which is typically characterized by the secretion of α-SMA and its further internalization to form stress fibers and the production of collagen I. The expression of α-SMA by renal tubular epithelial cells during EMT is used to identify EMT by immunostaining, thus demonstrating the existence of this process (Strutz et al., 1995; Iwano et al., 2002; Cheng et al., 2006; Tyler et al., 2006).

### 2.1 TGF-β1: The central regulator of renal fibrosis

When the kidney is damaged, TGF-β1 is secreted into the renal interstitium by a variety of recruited inflammatory cells and almost all renal cells. TGF-β1plays a role in the development of renal fibrosis as a negative regulator of cells and tissue (Miyazono et al., 2001; Shi and Massagué, 2003). TGF-β1 is a crucial regulator of renal fibrosis. TGF-β1 synthesis usually takes the form of precursors and binds specifically to TGF-β binding protein. When the complex precursor is cleaved by proteases such as fibrinolytic enzymes or matrix metalloproteinases (MMP), the TGF-β1 precursor releases a latency-associated peptide (LAP) that is simultaneously separated from the TGF-β1 binding protein, at which point TGF-β1 is activated and binds to its receptor (Annes et al., 2003; Robertson et al., 2015). Inazaki et al. (2004) constructed a Smad3-deficient UUO mouse model and found that it displayed significantly reduced renal fibrosis compared to Smad3^+/+^ mice; the pathological manifestations were, in fact, comparable to those in wild-type mice, suggesting the importance of Smad3 in the signaling cascade response of TGF-β1. Based on existing work, it has been concluded that TGF-β1 induces the expression of target genes mainly through a Smad-dependent pathway (Smad-dependent) and a Smad-independent pathway (Smad-independent), and ultimately triggers renal fibrosis by activating myofibroblasts or causing excessive deposition of ECM. The classical TGF-β1/Smad pathway mainly binds TGF-β1R1 (ALK5) and TGF-β1R2 (BMPR-II) to phosphorylate further the regulatory R-Smad (Smad2 and Smad3), which forms a complex with the Co-Smad (Smad4) in the cytoplasm to ectopically initiate target gene transcription in the nucleus (Shi and Massagué, 2003; Meng et al., 2016). The independent Smad TGF -β1 pathway is mainly mediated through interaction with the three kinases of the mitogen-activated protein kinase (MAPK) family, p38, c-Jun terminal kinase (JNK), and extracellular signal-regulated kinase (ERK); numerous studies have shown that all three MAPKs are activated in chronic kidney injury (Adhikary et al., 2004; Stambe et al., 2004a; De Borst et al., 2007). These three MAPKs phosphorylate three serine (S) and one threonine (T) residues in the Smad2/Smad3 linkage region, thereby promoting the classical Smad pathway-dependent signaling of TGF-β1 (Kamato et al., 2013). It has been demonstrated in animal models that inhibition of p38, or blockade of JNK/c-Jun signaling, can effectively inhibit the progression of renal fibrosis (Stambe et al., 2004b; Ma et al., 2007; Ma et al., 2009; Müller et al., 2013). This suggests that, in renal fibrosis mediated by TGF-β1 as a central regulator, there exist highly complex interactions involving multiple signaling factors and their expression products. Our current understanding of these signaling mechanisms remains inadequate.

### 2.2 Extracellular matrix deposition

ECM is divided into two main categories: one is mesenchymal connective tissue with extracellular scaffolding function related to physiological activity, and the other is related to the formation of fibrosis, mainly composed of collagen, Laminins, fibronectin, and several Proteoglycans (Bonnans et al., 2014; Raeeszadeh-Sarmazdeh et al., 2020), Recent studies have shown that ECM is mainly produced by three functional areas of the kidney, specifically the glomeruli, tubules, and blood vessels, which participate in the production and degradation of interstitial ECM. Myofibroblasts are the most critical and are activated under pathological conditions, secrete large amounts of ECM, and exacerbate the development of renal fibrosis (Rayego-Mateos et al., 2021).

The deposition of ECM is an essential component of renal fibrosis pathology. The process is a double-edged sword in the development of renal fibrosis. On the one hand, the deposition of ECM accelerates the process of renal fibrosis. On the other hand, ECM interacts with cytokines and growth factors to enhance or inhibit renal fibrosis. ECM production is accompanied by the release of many soluble molecules, which directly or indirectly bind Toll-like receptors (TLR2/4) expressed on immune cells, and further activate the NF-κB signaling pathway to mediate the production of various cytokines and chemokines, forming a pro-fibrosis vicious cycle. Conversely, the soluble core can inhibit the TGF-β1 signaling pathway by inhibiting apoptosis of residual renal tubular epithelial cells by binding to insulin-like growth factor receptor (IGF-1) and increasing fibroblast production of Fibrillin 1. It can also inhibit CTGF-mediated production of collagen III and fibronectin in fibroblasts, thus exerting antifibrotic effects (Nastase et al., 2018).

### 2.3 The triggering of epithelial-mesenchymal transition

There is ongoing debate as to whether EMT occurrs during renal fibrosis. Lineage tracing studies have demonstrated that EMT does not occur *in vivo*. However, type 2 EMT has been suggested to play an important role in fibrosis, with renal tubular epithelial cells acquiring a mesenchymal fibroblast-like phenotype and secreting extracellular matrix such as collagen fibers (Marconi et al., 2021). Zeisberg et al. (Zeisberg and Kalluri, 2004; Zeisberg and Kalluri, 2008) summarized the importance of EMT involvement in renal fibrogenesis. Firstly, TGF-β1 was shown to couple with the type I receptor ALK-5 and type II receptor serine/threonine kinase receptor (BMPR-II) in renal tubular epithelial cells to further phosphorylate and activate Smad2/3. This activated Smad2/3 further bound to Smad4 and translocated into the nucleus, inducing transcriptional effects. At the same time, TGF-β1 can also enhance the Smad pathway through the JNK and RhoA pathways. The TGF-β1-mediated signaling pathway triggers a series of morphological transformations such as the formation of fibrous bundles in the renal tubular epithelium, the reduction or even disappearance of the apical brush border (likely due to TGF-β1-mediated downregulation of E-cadherin), the destruction of the basement membrane, and the disappearance of tight junctions between the epithelium.

### 2.4 Infiltration of immune cells promote renal fibrosis

During renal tissue repair, multiple immune cells are involved in the inflammatory and fibrotic processes. Macrophage polarization plays an important role in chronic kidney injury, mainly involving the infiltration of M1 macrophages in the early stages of kidney injury in response to inflammation and cellular damage. In the late stages, the infiltration of M2 (M2b, M2C) macrophages occurs as part of the tissue repair process. In chronic kidney disease, macrophages become polarized from the M1 to M2 phenotype by pro-fibrotic factors such as TGF-β1, and their massive infiltration into the renal interstitium promotes the deposition of ECM. At the same time, their secreted cytokines, such as IL-1, MMP (2, 9, 12), platelet-derived growth factor, and other regulatory factors, further promote the proliferation and activation of fibroblasts, which intensify the progression of renal fibrosis (Tang et al., 2019). Zhang et al. reported that various T cell subsets mediate glomerular injury and pro-fibrotic mechanisms during renal inflammation, with tissue-associated γδ T cells playing an important role in the development of renal interstitial fibrosis (Zhang and Zhang, 2020). γδ T cells further activate myofibroblasts and promote ECM deposition by producing IL-17A in the renal interstitium in chronic kidney disease (Peng et al., 2015; Law et al., 2019). CD4^+^T lymphocytes can produce TGF-β1, thereby modulating myofibroblasts, and can also promote the secretion of PDGF, TGF-β1, CTGF, fibroblast growth factor-2 (FGF-2), and other cytokines and growth factors by activating macrophages or renal tubular epithelial cells to induce the proliferation and activation of fibroblasts, ultimately resulting in the deposition of ECM (Nikolic-Paterson, 2010).

### 2.5 Bone morphogenetic protein-7: The protective regulator of renal fibrosis

Bone morphogenetic protein-7 (BMP-7), a member of the transforming growth factor TGF -β superfamily, reverses TGF-β1-induced EMT and epithelial cell injury in a mouse model of chronic kidney injury. BMP-7 activates Smad5 by coupling to the type I receptor ALK-3 and the type II receptor (BMPR-II) of renal tubular epithelial cells. The phosphorylated Smad5 further binds to Smad4, causing upregulation of E-cadherin and intercellular tight junction protein (ZO-1) expression, resulting in the reversal of renal fibrosis (Zeisberg et al., 2003; Zeisberg and Kalluri, 2004).

### 2.6 G2/M phase block

Li et al. (Yang et al., 2010) constructed three models of renal injury, namely ischemia-reperfusion injury (IRI), aristolochic acidosis (AAN), and unilateral ureteral ligation (UUO), and found a significant correlation between the development of renal fibrosis and the arrest of renal tubular epithelial cells in the G2/M cell cycle DNA damage checkpoint phase. When renal tubular epithelial cells are damaged, a large number of renal epithelial cells become arrested in the G2/M phase *vi*a the activation of the protein kinases Chk1 and Chk2, which are downstream of the ATM-ATR signaling pathway. At the same time, the G2/M phase arrest activates the JNK signaling pathway, which mediates the production of many pro-fibrotic factors, such as TGF-β and CTGF, thus promoting the development of renal fibrosis.

### 2.7 Other mechanisms

*p53*, a tumor suppressor gene, also plays an essential regulatory role in renal fibrosis. TGF-β can activate serine protein kinase (ATM) through phosphorylation of ROS signaling, which allows activation of p53 phosphorylation and promotes the formation of the p53/Smad3 complex, which translocates to the nucleus to induce transcription of fibrogenic factors (Meng et al., 2016).

Renal pathological biopsies from ESRD and various renal diseases show that renal fibrosis is associated with elevated connective tissue growth factor (CTGF) expression, and is associated with elevated TGF-β1. It was found that the CTGF mainly originated from α-SMA-positive myofibroblasts, suggesting that myofibroblasts synthesize CTGF by autocrine or paracrine means following induction by TGF-β1. The elevated CTGF promotes the development of renal interstitial fibrosis by increasing the phosphorylation of R-Smad (Smad2/Smad3) and promoting TGF-β1 signaling (Toda et al., 2018). CTGF may be involved in the G2/M phase arrest of renal tubular epithelial cells, indirectly promoting the development of fibrosis (Yang et al., 2010).

Mammalian target of rapamycin (mTOR), a serine/threonine-protein kinase, can be activated during kidney damage. Current studies suggest that TGF-β helps protect mTOR from degradation by inhibiting Deptor (an inhibitor of mTOR) in a Smad-dependent manner, and also activates mTOR1 and mTOR2 by inducing the PI3K/AKT signaling pathway in renal fibrosis (Lieberthal and Levine, 2009; Lieberthal and Levine, 2012; Das et al., 2013).

The epidermal growth factor receptor (EGFR) is a tyrosine kinase that binds to EGF and can specifically phosphorylate and activate Smad3 (Meng et al., 2016). In cultured renal mesenchymal fibroblasts, EFGR was found to promote EMT formation through activation of STAT3 and the ERK (1/2) pathway (Liu et al., 2012). TGF-β1 can also activate EFGR *via* tyrosine kinase Src; blocking of c-Src was found to reduce EFGR and TGF-β1 activation and thus renal fibrosis injury in an animal model of UUO (Samarakoon et al., 2013; Yan et al., 2016).

The Wnt/β-catenin signaling pathway can be activated during renal injury and promotes the expression of many genes associated with fibrosis such as Snail 1, Fibrinogen activator inhibitor (PAL-1), and MMP-7, which promote the development of fibrosis (Hao et al., 2011; Tan et al., 2011). The Wnt-specific antagonist Dickkopf-1 significantly inhibits the accumulation of β-catenin and suppresses the expression of pro-fibrotic factors and the activation of myofibroblasts (He et al., 2009). Wnt/β-catenin may potentially interact with TGF-β/Smad in renal fibrosis, however, the specific intrinsic association between the two pathways needs to be elucidated by further experimental studies.

## 3 Histone deacetylases regulate renal fibrosis

The histone deacetylase family mediates chromatin compression by deacetylating histones inside the cell, ultimately repressing gene transcription. In contrast, histone acetyltransferases (HATs) promote gene expression by acetylating histones and thereby spreading chromatin. However, it has been shown that HDACs can deacetylate and modulate cytokines other than histones to produce various biological effects (Chen et al., 2001; Hubbert et al., 2002; Simonsson et al., 2005).

Histone deacetylases are typically categorized into four classes: class I (HDAC1, 2, 3, and 8), class II containing class IIa (HDAC4, 5, 7, 9) and class IIb (HDAC6, HDAC10), class III (sir-like proteins; Sirt1-7), and class IV is the recently discovered HDAC11. Activating class III HDAC molecules requires nicotinamide adenine dinucleotide (NAD^+^) as a cofactor. In contrast, the activation of the other three HDAC classes requires Zn^+^ as a cofactor. Notably, class I HDACs are mainly expressed in the kidney (de Ruijter et al., 2003).

HDACs are important epigenetic regulators that play an essential role in various renal diseases such as polycystic kidney disease, renal cell carcinoma, podocyte injury, and renal fibrosis (Liu, 2021). HDACs may act through different signaling pathways and produce different disease progression mechanisms. We summarize how HDACs promote renal fibrosis according to different HDACs subtypes in Table 1.

**TABLE 1:** Preclinical effects of HDAC superfamily regulate renal fibrosis.

Class	Damage/disease model	Targets	Mechanism	References
HDAC1, 2, 8↑	HMC	TGFβ1/Smad and JAK2/STAT3	HDAC1, 2, 8 activate TGF-β1/Smad2/3 and Jak2/Stat3 signaling pathways, promoting HMC proliferation and ECM deposition	Dai et al. (2016)
HDAC1, 2↑	*Npr1* haplotype male mice	STAT1	HDAC1 and HDAC2 inhibit STAT1 from binding to NF-κB to form a complex by deacetylating STAT1	Kumar et al. (2017)
HDAC3↑	UUO	Klotho↓	HDAC3 represses klotho expression by recruiting NCoR and NF-κB to form a complex that acts on the klotho promoter	Chen et al. (2021)
	AAN1			
HDAC3↑	Adenine CKD	PPARγ	HDAC3 inhibits klotho protein-associated transcription factor PPARγ acetylation, thereby suppressing klotho protein expression	Lin et al. (2017)
HDAC3↑	FSGS	miR-30d↓	HDAC3 in combination with Smad2/3 and NcoR form a repressor complex that acts in the vicinity of the miR-30d promoter and regulates the downregulation of miR-30d	Liu et al. (2016)
HDAC3↑	UUO	TIMAP↓	HDAC3 inhibite the TGF-β-HDAC3/Smad-TIMAP pathway and enhance the activity and phagocytosis of M2-type macrophages	Yang et al. (2017)
HDAC8↑	UUO	EMT	HDAC8 can arrest renal tubular epithelial cells in G2/M phase and subsequently promote EMT.	Zhang et al. (2020)
HDAC4↑	UUO	TGFβ1/Smad	HDAC4 enhances the TGF-β1/Smad pathway and promotes the deposition of ECM.	Xiong et al. (2019)
		NF-κB↑		
		Klotho↓		
		BMP-7↓		
HDAC4	UUO	p38↑	HDAC4, HDAC5 promote p38-MAPK pathway	Choi et al. (2016)
HDAC5 ↑				
HDAC7 ↑	Human lung fibroblasts	PGC1α	HDAC7 deacetylates histone H3 near the promoter of the anti-fibrotic gene PGC1α, producing a suppressive effect on the expression of anti-fibrotic genes such as PGC1α and ultimately promoting fibroblast activation and proliferation	Jones et al. (2019)
	Peyronie’s disease (PD) fibroblast	TGFβ1/Smad	HDAC7 promotes the TGF-β1/Smad pathway and myofibroblast activation	Kang et al. (2018)
HDAC9 ↑	Endothelial cells	p38/MAPK	HDAC9 can promote vascular endothelial cell injury by acting on the p38 MAPK pathway	Kuang et al. (2021)
HDAC6↑	ANGII induced hypertensive model	Smad2/3	HDAC6 promotes the activation of Smad2/3 phosphorylation as well as increases the binding activity of R-Smad to the promoter of pro-fibrotic genes	Choi et al. (2015)
HDAC1, 4, 5, 6, 10 ↑	UUO	p38/MAPK	HDAC1, 4, 5, 6, 10 act on p38/MAPK pathway	Choi et al. (2016)
HDAC8↓				
SIRT1↑	TGF-β receptor overexpression	TGF-β/Smad	SIRT1 enhances TGF-β-mediated release of ECM.	Zerr et al. (2016)
SIRT1	UUO	Smad3	SIRT1 can deacetylate Smad3, resulting in antifibrotic effects	Li et al. (2010)
SIRT2↑	UUO	MDM2↑	SIRT2 upregulates MDM2, activates p53 and inhibits its transcriptional activity to promote myofibrillar activation	He et al. (2018)
SIRT1↑	UUO	PCNA↑ cyclin↑	SIRT1 and SIRT2 increase the expression of PCNA, cyclin D1 and cyclin E, which promote the activation and proliferation of fibroblasts	Ponnusamy et al. (2014)
SIRT2↑				
SIRT3	ANGII induced hypertensive model	pericytes	SIRT3 inhibits the conversion of pericytes to fibroblasts	Feng et al. (2020)
	CKD	mitochondria	Mito-TEMPO can alleviate mitochondrial dysfunction through the SIRT3-SOD2 pathway	Liu et al. (2018)
SIRT4	—	NAD^+^, AMPK	SIRT4 has regulatory effects on various factors including NAD^+^, AMPK.	Mazumder et al. (2020)
SIRT5	—	Mitochondria & Peroxisomes	SIRT5 regulates fatty acid oxidation homeostasis between mitochondria and peroxisomes in proximal renal tubular epithelial cells	Chiba et al. (2019)
SIRT6	RTECs	β-catenin	SIRT6 binds to β-catenin and mediates histone deacetylation near the promoters of fibronectin, MMP7, and snail	Cai et al. (2020); Gewin (2020)
	Calorie restriction mouse	NF-κB	SIRT6 inhibits NF-κB signaling, a nuclear factor associated with inflammation and aging	Zhang et al. (2016)
SIRT7	—	TGFβ1/Smad	SIRT7 inhibits the TGF-β1/Smad signaling pathway and regulates fibrosis by deacetylating Smad4 and reducing Smad3 levels	Chen et al. (2017); Li et al. (2022)
HDAC11 ↑	RTECs	*KLF15* promoter	Formation of HDAC11- AP-2α complex suppressed KLF15 mRNA and protein levels, resulting in increased expression of pro-fibrotic factors	Mao et al. (2020)

### 3.1 Class I histone deacetylases

Dai et al. treated human mesangial cells (HMC) *in vitro* with isolated poly IgA1 (P-aIgA1) and found that HDAC1, HDAC2, and HDAC8 upregulated the expression and subsequent activation of TGF-β/Smad2/3 and Jak2/Stat3 signaling pathways, which promoted the proliferation of HMCs and facilitated the production of extracellular matrix deposition, such as collagen I. In addition, activation of the TGF-β1/Smad pathway induced the proliferation and activation of myofibroblasts and promoted the progression of renal interstitial fibrosis (Dai et al., 2016). Utilizing a diabetic mouse model, Ma et al. found that short-chain fatty acids (SCFAs) enhance the autophagic effect by inhibiting HDAC2, deregulating the inhibitory effect of HDAC2 on the autophagy-activated kinase ULK1 promoter, and then upregulating ULK1, thereby reducing renal fibrosis progression (Ma and Wang, 2022), In addition, in Npr1 (coding for receptor guanylyl cyclase-A [GC-A/NPRA]) haplotype male mice, HDAC1 and HDAC2 inhibit STAT1 binding to NF-κB by deacetylating STAT1, which leads to enhanced NF-κB binding activity to pro-inflammatory and pro-fibrotic genes, thereby promoting the progression of renal fibrosis (Kumar et al., 2017). In the 1990s, researchers extracted cDNA encoding HDAC3 from humans. Analysis of the amino acid sequence encoded by the open reading frame (ORF) of the HDAC3 gene revealed that its gene homology with HDAC1 and HDAC2 is around 50% (Yang et al., 1997; Mahlknecht et al., 1999).

Chen et al. (2021) constructed renal fibrosis models by using UUO and AAN. HDAC3 was found to be highly expressed in the mouse model, accompanied by low expression of the anti-aging protein klotho. After knocking down the HDAC3 gene, there was no significant difference in the expression of klotho between the experimental and sham-operated groups. The application of the TGF-β receptor inhibitor SB431542 to UUO mice revealed that it alleviated renal fibrosis. When the Smad3 inhibitor (SIS3) was added, HDAC3 expression was found to be reduced, thus indicating that the TGF-β/Smad3 signaling pathway upregulated HDAC3. Meanwhile, HDAC3 formed a complex by recruiting NCoR and NF-κB, which acted on klotho. In addition, HDAC3 inhibited the expression of klotho by recruiting NCoR and NF-κB to form a complex that acts on the klotho promoter, thereby promoting the progression of renal fibrosis, developing a pro-fibrotic pathway initiated by TGF-β with HDAC3 as an intermediary.

In a mouse model of chronic kidney disease utilizing adenine administration, histone deacetylase 3 (HDAC3) inhibits the acetylation of the klotho protein-associated transcription factor PPARγ, suppresses klotho protein expression and reduces secretory klotho concentrations in the blood, thereby increasing the progression of chronic kidney disease (Lin et al., 2017). In patients with focal segmental glomerulosclerosis (FSGS), there is significantly downregulated expression of the microRNA-30 family, which maintains glomerular homeostasis. *In vitro* studies revealed that elevated HDAC3, under the regulation of TGF-β, combined with Smad2/3 and NcoR to form an inhibitory complex near the miR-30d promoter. The final negative regulation of downregulated miR-30d, which aggravates the glomerular injury, will further exacerbate the renal injury. TSA (trigonellin A) and RGFP966 can alleviate the complex-mediated downregulation of these miRNA levels that maintain glomerular homeostasis, thus exerting a protective effect on the kidney (Liu et al., 2016).

Some investigators have demonstrated that HDAC3 creates a transcriptional repressor complex with SMRT and NcoR by successfully isolating the respective enzymatically active complex and demonstrating the interaction (Li et al., 2000; Wen et al., 2000). These studies suggest that the HDAC3 complexes may collaborate to negatively regulate organism functions. HDAC3 regulates TIMAP (TGF-β inhibited membrane-associated protein), a protein downstream of TGF-β that is enriched on the cell membrane surface. In renal fibrosis mice, TIMAP expression was shown to be reduced, likely due to the elevated expression of TGF-β, which promoted the high expression of HDAC3 and activated the Smad signaling pathway, which combined with HDAC3 to bind near the TIMAP promoter and inhibit the expression of TIMAP, which could dephosphorylate the light chain of myosin in M2 macrophages, thereby reducing their migration and phagocytosis (Yang et al., 2017). The activation of TGF-β and the M2 macrophage polarization promote the development of renal fibrosis (Mosser and Edwards, 2008; Casalena et al., 2012). This suggests that elevation of TGF-β enhances the activity and phagocytosis of M2 macrophages. However, this may further aggravate tissue damage and the progression of chronic disease. Therefore, inhibition of the TGF-β-HDAC3/Smad-TIMAP pathway may slow down the progression of TGF-β-mediated renal fibrosis.

**FIGURE 2:**
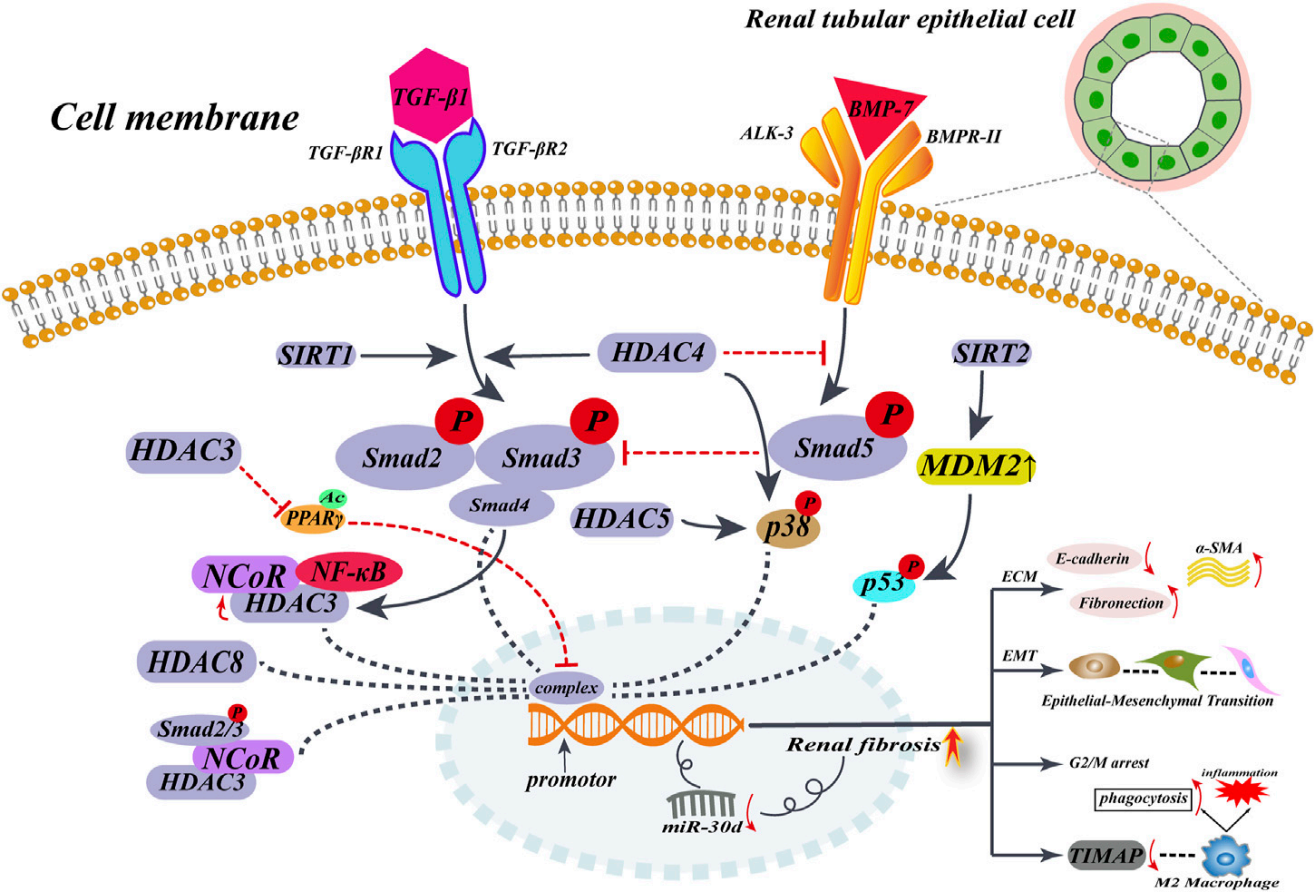
HDACs promote renal interstitial fibrosis in non-histone epigenetic modalities of regulation. When renal fibrosis begins, TGF-β expression is upregulated, and elevated TGF-β upregulates HADC expression in renal tubular epithelial cells, in a Smad2/3-dependent way. Then, HDACs translocate into the nucleus by recruiting multiple cytokines to form complexes that act near the gene promoter, mediating the expression of α-SMA and collagen I. On the other hand, elevated HDACs inhibit the anti-fibrotic factor BMP-7/Smad5 pathway and deregulate the protective factor of BMP-7, ultimately promoting the development of renal fibrosis.

The effect of HDAC8 on renal fibrosis, another important subtype of the type I HDAC family, has also been investigated. It was found that HDAC8 was highly expressed in the UUO kidney model and that HDAC8 specific selective inhibitor PCI34051 or silencing HDAC8 by siRNA alleviated fibrosis progression and inhibited EMT triggered by TGF-β, and that high HDAC8 expression in UUO kidneys could cause pro-fibrotic factors to be produced. Most importantly, elevated HDAC8 arrests renal tubular epithelial cells in the G2/M phase, which promotes the production of the pro-EMT factor (Snail), which is necessary to initiate EMT. However, the application of HDAC8 selective inhibitors can alleviate the EMT process. HDAC8 inhibitors may also reverse the decrease of BMP-7 and klotho, exerting an anti-fibrotic effect (Figure 2) (Zhang et al., 2020).

### 3.2 Class IIa histone deacetylases

Xiong et al. (2019) examined the effect of the selective class IIa HDACs inhibitor MC1568 on renal fibrosis with UUO. They found that renal injury was followed by a significant elevation of class IIa HDACs, HDAC4 was most significantly upregulated in renal tubular epithelial cells. Elevated class IIa HDACs enhanced the TGF-β1/Smad pathway and promoted the deposition of collagen I, α-SMA, and fibronectin. Meanwhile, class IIa HDACs enhanced the activation of phosphorylation of inducible transcription factor (NF-κB) and promoted the production of degradative enzymes with ECM degradation function, namely MMP-2 and MMP-9. In addition, they inhibited the down-regulation of two renal fibrosis protective factors (klotho and BMP-7). When the fibrotic mice were treated with MC1568 or siRNA, the above metabolic activities were inhibited and the production of MMP-2 and MMP-9 was enhanced, helping to degrade the extracellular matrix and reducing the progression of renal fibrosis. A study using a mouse model of Alport syndrome, which inhibits metalloproteinase (MMP) at different stages of renal disease, seems to reveal a pattern whereby elevated MMP promotes EMT and the progression of fibrosis in its early stages. In contrast, elevated MMP plays a role in degrading the extracellular matrix in the late stages of renal fibrosis, mitigating renal fibrosis (Zeisberg et al., 2006). Piceatannol, a phenolic compound, inhibits the expression of HDAC4 and HDAC5 in unilateral ureteral obstruction UUO and may also inhibit the p38-MAPK pathway to produce antifibrotic effects (Choi et al., 2016). The mechanism by which HDAC7 promotes fibrosis in renal fibroblasts has not been extensively studied. When TGF-β was applied to human lung fibroblasts, it was found that TGF-β, through the intermediate regulator HDAC7, deacetylates histone H3 near the promoter of the anti-fibrotic gene PGC1α, inhibiting the expression of anti-fibrotic genes such as PGC1α, thereby ultimately promoting the activation and proliferation of fibroblasts and causing massive deposition of ECM such as collagen I and fibronection (Jones et al., 2019). In Hepatic Stellate cell (HSC) cells, cytokines such as IL-6 and TNF promote the binding of HDAC7 to the anti-tumor gene cylindromatosis (CYLD), which recruits HDAC7 to the nucleus near the promoter of the hepatocyte growth factor (HGF). In the absence of CYLD, HGF expression is downregulated, ultimately triggering hepatocyte injury and promoting the progression of liver fibrosis (Pannem et al., 2014). In Peyronie’s disease (PD), the fibroblast expression of HDAC7 was significantly higher than normal. When HDAC7 was knocked down, the TGF-β1/Smad pathway was inhibited and the phosphorylation-activated Smad2/3 complex was unable to enter the nucleus, inhibiting myofibroblast activation, which reduced the deposition of extracellular matrix such as collagen I (Kang et al., 2018). This suggests that HDAC7 plays an important role as an intermediate factor in the progression of fibrosis. The correlation between HDAC9 and renal fibrosis is less studied, however, HDAC9 has been shown to promote vascular endothelial cell injury by acting on the p38 MAPK pathway (Kuang et al., 2021).

### 3.3 Class IIb histone deacetylases

In a mouse model of hypertension generated using Angiotensin II (ANGII), Choi et al. (2015) demonstrated that the HDAC6 selective inhibitor (Tubastatin A) combined with a Smad3 knockdown inhibited TGF-β and ANG-induced renal fibrosis. It was shown that HDAC6 promotes the activation of Smad2/3 phosphorylation as well as increases the binding activity of R-Smad to the promoter of pro-fibrotic genes, increasing the expression of ANGII-induced collagen I, collagen III, fibronectin, and other extracellular matrices, as well as the expression and release of inflammatory factors such as monocyte chemotactic protein-1 (MCP-1) and fibrinogen activator inhibitor-1 (PAI-1). TubA or knockdown of HDAC6 inhibits the secretion of these inflammatory factors. At the same time, HDAC6 also enhances the binding activity of histone H4 to the collagen I promoter and promotes the deposition of type I collagen In the renal interstitium. HDAC6 has been found to play an important regulatory role in TGF-β-induced EMT. TGF-β increases the activity of HDAC6, and activated HDAC6 deacetylates the renal tubular epithelial cytoskeletal protein (α-tubulin). The acetylation of α-tubulin can be significantly upregulated, accompanied by a decrease in the degree of EMT. α-tubulin deacetylation modifications promote cytoskeletal reorganization during EMT formation (Shan et al., 2008; Gu et al., 2016). How TGF-β activates HDAC6 is not known. Perhaps the deacetylation modification of α-tubulin by HDAC6 provides the structural basis for EMT. In combination with the genetic reprogramming of renal tubular epithelial cells in response to TGF-β/Smad signaling, it leads to EMT, which may also be present during EMT in renal fibrosis. In UUO mice, HDAC1, 4, 5, 6, 10 upregulated expression and HDAC8 downregulated expression, which may exert pro-fibrotic effects through a TGF-β1/Smad independent pathway (Choi et al., 2016). However, the mechanism of action of class IIb HDACs in the fibrosis process still needs to be further explored.

### 3.4 Class III histone deacetylases

The class III HDAC family members currently thought to be associated with the development of renal fibrosis mainly include SIRT1 and SIRT2; there is no consensus on the role of SIRT1 in renal fibrosis. SIRT1 is localized in the nucleus, and its activation is dependent on NAD^+^ as a coenzyme; resveratrol is an activator of SIRT1. Zerr et al. (2016) found in a mouse model of bleomycin-induced skin fibrosis and TGF-β receptor overexpression that SIRT1 was reduced under the regulation of overactivated TGF-β. The activation of SIRT1 enhances TGF-β-mediated extracellular matrix release and exacerbates the extent of the fibrotic response. Specific knockdown of the SIRT1 gene inhibits the TGF-β/Smad signaling pathway, and reduces Collagen release from fibroblasts and the fibrotic response. However, in a UUO mouse model, some investigators found that acetylation of Smad3 under the regulation of TGF-β appeared to mediate the renal fibrotic process, and SIRT1 could deacetylate Smad3, resulting in antifibrotic effects. Resveratrol produced antifibrotic effects by activating SIRT1, reducing the activation of myofibroblasts, the deposition of ECM, and other fibrotic responses (Li et al., 2010). These differences may be attributed to the different roles of SIRT1 in renal fibrosis. SIRT2 is localized in the nucleus. Its expression is significantly increased in the renal interstitium of fibrotic patients and the UUO mouse model. SIRT2 expression is elevated upon induction of TGF-β1 in fibroblasts. It promotes myofibroblast activation, as well as α-SMA collagen III, fibronectin, and other extracellular matrix deposition, a process that is significantly inhibited by applying the SIRT2-specific inhibitor AGK2. However, SIRT2 was not found to affect renal tubular epithelial-to-mesenchymal transition. At the same time, murine double minute2 (MDM2) was also found to be induced by TGF-β1 and may be involved in myofibrillar activation through activation of P53 and inhibition of its transcriptional activity. Inhibition of SIRT2 was followed by downregulation of MDM2. In contrast, inhibition of the SIRT-MDM2 interaction did not alter the TGF-β1-mediated increase in SIRT2 expression, suggesting that SIRT2 may be an upstream regulator of MDM2 and that it exerts a pro-fibrotic effect through MDM2 (He et al., 2018).

Ponnusamy et al. (2014) reported that the addition of SIRT1 and SIRT2 inhibitors (Sirtinol) or knockdown of SIRT1 and SIRT2 by siRNA in UUO mice significantly decreased proliferating cell nuclear antigen (PCNA) and cell cycle proteins (cyclinD1, cyclinE), which in turn inhibited fibroblast activation and proliferation, and inhibited α-SMA, collagen I, fibronectin, and other extracellular matrix deposition. When applied alone, the SIRT1 inhibitor EX527 or the SIRT2 inhibitor AGK2 was found to produce similar antifibrotic effects in cultured NRK-49F rat fibroblasts. In conclusion, there may be a complex regulatory network of SIRT1,2 involved in the development of renal fibrosis. Its pro-fibrotic mechanisms need to be further explored.

SIRT3 is mainly localized in the mitochondrial matrix and is the main regulator of organelle deacetylation. Marina Morigi summarized the regulatory mechanisms of different isoforms of SIRTs in renal physiology. SIRT1 and SIRT3 can exert protective effects against renal fibrosis by inhibiting inflammatory responses and apoptosis, and by regulating energy metabolism (Morigi et al., 2018). In the Ang II-induced hypertension mouse model, SIRT3 knockdown enhances the conversion of pericytes to fibroblasts, while inducing iron overload, decreasing glucose metabolism in cells, and causing ROS production, thus causing kidney tissue damage and aggravating the progression of renal fibrosis (Feng et al., 2020). The role of SIRT3 in deacetylation of pyruvate dehydrogenase E1α (PDHE1α) is closely related to metabolic reprogramming during renal fibrosis (Zhang et al., 2021). In CKD mice, mitochondria-targeted superoxide mimetic (Mito-TEMPO) can protect against renal fibrosis by slowing mitochondrial dysfunction and endoplasmic reticulum stress through the SIRT3-SOD2 pathway (Liu et al., 2018). The regulatory mechanisms of SIRT4 in renal fibrosis have not been fully established. SIRT4 acts as a deacetylase associated with the cell stress response. In metabolic reprogramming, it regulates various factors such as NAD^+^ and ATP-dependent protein kinase (AMPK), and exerts antioxidant damage and repair effects. SIRT4 can also regulate myofibroblast transdifferentiation and subsequent collagen fiber deposition by regulating glutamine (Gln) metabolism. SIRT4 can also promote fibrosis progression by increasing ROS levels in cardiac myocytes and sensitivity to Ang II responses to promote fibrosis progression (Luo et al., 2017; Mazumder et al., 2020). SIRT5 is also a mitochondria-localized deacetylase but few studies have been published on its regulatory mechanisms associated with chronic kidney disease and renal fibrosis. However, SIRT5 can reduce AKI injury by regulating the balance of mitochondrial and peroxisomal fatty acid oxidation in proximal renal tubular epithelial cells (Chiba et al., 2019). In renal tubular epithelial cells, SIRT6 binds to β-catenin and mediates histone deacetylation near the promoters of fibronectin, MMP7, and snail, exerting a protective effect against renal fibrosis by inhibiting their transcription (Cai et al., 2020; Gewin, 2020). In a caloric restriction (CR) mouse model, SIRT6 slows the development of renal fibrosis by inhibiting NF-κB signaling, which in turn slows the proliferation and senescence of WI38 fibroblasts (Zhang et al., 2016). SIRT7 has been shown to inhibit the TGF-β1/Smad signaling pathway and regulate fibrosis by deacetylating Smad4 and reducing Smad3 levels. Since the Wnt/β-catenin pathway plays a key role in the development of fibrosis, SIRT7 may regulate fibrosis progression by inhibiting the Wnt/β-catenin pathway (Chen et al., 2017; Li et al., 2022). However, our understanding of the specific regulatory mechanisms of SIRT7 in renal fibrosis is limited and this topic calls for significant research attention.

### 3.5 Class IV histone deacetylases

In renal tubular epithelial cells (RTECs), HDAC11 expression was upregulated after administration of Ang II; HDAC11 formed a complex with Activator protein 2 (AP-2α). Kruppel-like factor 15 (KLF15), a regulator of renal fibrosis, may play an anti-fibrotic role by inhibiting ERK/MAPK, TNK/MAPK, Wnt/β-catenin, and other pathways. Since the vicinity of the KLF15 promoter may contain AP-2α binding sites, the formation of the HDAC11- AP-2α complex inhibits KLF15 mRNA and protein levels, and the expression of collagen I, α-SMA, and other genes was increased, thus promoting the progression of renal fibrosis (Mao et al., 2020).

### 3.6 Histone H3 deacetylation mediates myofibroblast activation and pro-fibrotic gene expression

Smith et al. (2019) utilized an *in vitro* culture of fibroblasts from a UUO rat model, to show that TGF-β can reduce the acetylation of histone H3 in fibroblasts, resulting in the activation of multiple pro-fibrotic genes. This may arise from a reduced expression of histone acetylases (HAT) and elevated levels of histone deacetylases (HDACs), and reduced availability of Acetyl CoA in fibroblasts. The imbalance of H3 acetylation could be corrected *via* exogenous administration of citrate or the pan-HDAC inhibitor TSA, reducing the activation of myofibroblasts. In a rat fibroblast model (NRK-49F), histone H3 in the vicinity of the promoter of the Fibrogenic gene can be modified by TGF-β-induced deacetylation, a regulatory process that can be inhibited by the class I HDAC inhibitor valproic acid (VPA), thereby reducing myofibroblast activation and proliferation as well as the accumulation of α-SMA, collagen I, and fibronectin (Nguyễn-Thanh et al., 2018).

## 4 Protective and reversal effects of histone deacetylases inhibitors on renal fibrosis

Previous studies have shown that aberrant acetylation and deacetylation of histones play an essential role in tumor proliferation and differentiation. Johnstone (2002) proposed that HDACi could inhibit the G2 to M cell cycle transition in tumor cells and induce a high expression of major histocompatibility complex (MHC) class I and II molecules, Intercellular Adhesion Molecule 1 (ICAM 1), and activating factor (CD40) in tumor cells, thereby increasing their immunogenicity and thus enhancing the body’s immune response to the tumor cells. The FDA has approved two non-specific HDAC targeting inhibitors, Vorinostat and Romidepsin, primarily for the treatment of cutaneous carcinoma and peripheral T-cell lymphoma (West and Johnstone, 2014). Bush and McKinsey (2010) suggested that the regulation of histone and non-histone deacetylation plays a critical epigenetic role in the pathogenesis of the renal disease. HDAC inhibitors can target a variety of HDAC-mediated transcription-related renal dysfunctions, such as EMT, aberrant activation of fibroblasts, ECM deposition, etc. Liu et al. (Liu and Zhuang, 2015) reported that HDAC inhibitors can delay the progression of renal fibrosis, reduce the formation of polycystic kidney disease, improve the condition of diabetic nephropathy, lupus nephropathy, and aristolochic nephropathy, and even play an important role in the anti-rejection reaction of transplanted kidneys in animal models. Therefore, HDAC inhibitors may be used to inhibit interstitial fibrosis, slow down the progression of fibrosis in kidneys, and slow down renal failure. In Table 2, we summarize published animal model experiments, which were primarily performed utilizing broad-spectrum HDAC inhibitors, and the molecular mechanisms involved in the anti-fibrotic process of HDACi which may eventually help us attenuate the progression of end-stage renal disease (Table 2). We also summarized the inhibitory activity of HDAC inhibitors associated with the slowing of renal fibrosis against different subtypes of HDACs, expressed as half-inhibitory concentrations IC_50_ values (Table 3) (Novotny-Diermayr et al., 2010; Lauffer et al., 2013; Ho et al., 2020; Bondarev et al., 2021). In the available clinical trials, the application of HDACi can trigger the risk of cardiotoxicity, blood cell suppression, gastrointestinal reactions, and even thrombosis, etc. We summarized the various adverse effects of HDACi (Table 4) (Bondarev et al., 2021; Nanau and Neuman, 2013; Knipstein and Gore, 2011; Smolewski and Robak, 2017; Jo et al., 2022; O'Connor et al., 2015; Siegel et al., 2009; Chu et al., 2015; Van Veggel et al., 2018; Yagishita et al., 2019; Fahey et al., 2019).

**TABLE 2:** Reversal of renal fibrosis by HDAC inhibitors in different disease models.

HDACi	Concentration used	Damage/disease model	Targets	Mechanism	References
PA	0.1, 1, 2.5 nM	UUO	ClassI HDACs	VPA inhibit the aberrant expression of TGF-β and phosphorylation of Smad2/3, while upregulating Smad7.	Nguyễn-Thanh et al. (2018)
	300 mg/kg	DN	ClassI HDACs	VPA inhibit TGF-β, and similarly reduce deposition of CTGF, α-SMA, collagen I, and fibronectin	Khan et al. (2015)
MS-275	20 mg/kg	UUO	ClassI HDACs	MS-275 inhibite the expression of TGF-βR1, blocking the activation of phosphorylation of the downstream signal Smad3	Liu et al. (2013); Hyndman et al. (2019)
	20 mg/kg	IRI			
FK228	0.5 mg/kg	UUO	HDAC1 DAC2	FK228 inhibite the expression of the CyclinD1 in fibroblasts, preventing the transition from G1 to S phase of fibroblasts during proliferation	Yang et al. (2019)
CG200745	30 mg/kg	UUO	Pan-HDACs	CG attenuate the expression of TGF-β mRNA and phosphorylation of Smad2/3 in UUO mice	Choi et al. (2018)
	30 mg/kg	AS	Pan-HDACs	CG inhibit the RAS system-mediated inflammatory response and subsequent activation of TGF-β	Suh et al. (2020)
	5 mg/kg	Hypertension	Pan-HDACs	CG inhibit apoptosis in renal tubular epithelial cells	Bae et al. (2019)
SB939	75 mg/kg	NRK-49F	Pan-HDACs	SB939 inhibit phosphorylation of ERK, p38, PI3K/AKT.	Kang et al. (2017)
TSA	100, 300, 1000 nM	RPTEC	Pan-HDACs	TSA induce the expression of TGF-β1-resistant signaling factors: Id2, BMP-7 and E-cadherin, enhancing renal tubular homeostasis	Yoshikawa et al. (2007)
	0.5 mg/kg for mice and 100 nM for NRK-49F	UUO	Pan-HDACs	TSA inhibit the activation STAT3	Pang et al. (2009)
	1 mg/kg	UUO	Pan-HDACs	TSA promote the phenotypic transformation of macrophages M1-M2c, which in turn inhibite the progression of inflammation in damaged kidneys.	Tseng et al. (2020)
SFN	0.5 mg/kg	DN	HDAC2	SFN reactivate the BMP-7-Smad1/5/8 pathway	Kong et al. (2021)
MS-275	20 mg/kg	UUO	ClassI HDACs	MS-275 inhibit the activation STAT3	Liu et al. (2013)
NaBu	0.5 mg/kg	*Npr1*	Pan-HDACs	ATRA and NaBu can reduce the expression of pro-inflammatory and pro-fibrotic genes downstream of NF-κB by acetylating the transcription factor STAT1, which in turn promote the binding of STAT1 to NF-κB to form a complex	Kumar et al. (2017)

**TABLE 3:** IC_50_ values of HDAC inhibitors (Novotny-Diermayr et al., 2010; Lauffer et al., 2013; Ho et al., 2020; Bondarev et al., 2021).

HDACi	Drug names	HDAC subtype, IC_50_(nM)										
		1	2	3	8	4	5	7	9	6	10	11
MS-275	Entinostat	243	453	248	—	—	—	—	—	—	—	—
SAHA	Vorinostat	60	42	36	173	20	36	129	49	29	60	31
FK228	Romidepsin	1	1	1	>10^3^	647	>10^3^	>10^3^	>10^3^	226	1	11
PXD101	Belinostat	26	22	19	22	15	25	51	24	10	59	27
SB939	Pracinostat	49	96	43	140	56	47	137	70	>10^3^	40	93
TSA	Trichostatin A	1.8 nM for HDAC										
LBH589	Panobinostat	3	2	2	22	1	1	2	1	1	31	4

**TABLE 4:** The adverse reactions of HDAC inhibitors.

HDACi	Drug names	Adverse reactions	References
VPA	Valproic acid	Hepatotoxicity, mitochondrial toxicity, ammonal encephalopathy, allergic reaction syndrome, neurotoxicity, metabolic and endocrine system adverse reactions, and teratogenicity	Nanau and Neuman, (2013)
MS-275	Entinostat	Hypophosphatemia, hyponatremia, hypoproteinemia, diarrhoea, nausea, anorexia, headache, neutropenia, thrombocytopenia, leucopenia	Knipstein and Gore, (2011)
FK228	Romidepsin	Cardiotoxicity (asymptomatic arrhythmias, non-specific ST/T wave changes), gastrointestinal reactions, neutropenia, thrombocytopenia, tumour lysis syndrome	Smolewski and Robak, (2017); Bondarev et al. (2021)
CG200745	Ivaltinostat	thrombocytopenia, neutropenia, anorexia, rash, gastrointestinal reactions, pneumonia, fatigue	Jo et al. (2022)
PXD101	Belinostat	Gastrointestinal reactions, fatigue, anaemia, thrombocytopenia, neutropenia, dyspnoea, pneumonia, tumour lysis syndrome, liver failure, risk of cardiac abnormalities	Bondarev et al. (2021); O'Connor et al. (2015)
SAHA	Vorinostat	Gastrointestinal reactions, thrombocytopenia, fatigue, dehydration, anorexia, risk of thrombosis and embolism, risk of cardiac abnormalities	Siegel et al. (2009); Bondarev et al. (2021)
SB939	Pracinostat	Gastrointestinal reactions, thrombosis, fatigue, thrombocytopenia, cardiotoxicity (atrial fibrillation, prolonged QT interval)	Chu et al. (2015)
LBH589	Panobinostat	Severe diarrhoea, peripheral neuropathy, weakness, fatigue, gastrointestinal, neutropenia, thrombocytopenia, lymphopenia, cardiotoxicity (T-wave inversion, ST-segment depression, prolonged QT interval)	Van Veggel et al. (2018); Bondarev et al. (2021)
SFN	—	Gastrointestinal reactions (nausea, vomiting, heartburn)	Fahey et al. (2019); Yagishita et al. (2019)

### 4.1 Histone deacetylases inhibitors exert anti-renal fibrosis effects by counteracting TGF-β1/Smad and TGF-β1/non-Smad pathways

In the UUO mouse model, the class I HDACs inhibitor, Valproic acid (VPA), inhibited the aberrant expression of the pro-fibrotic signal TGF-β1 and subsequent phosphorylation of Smad2/3 while upregulating the protective cytokine Smad7 (Nguyễn-Thanh et al., 2018). VPA significantly ameliorated fibrosis in a diabetic rat model by inhibiting TGF-β1 and similarly reduced extracellular matrix deposition of CTGF, α-SMA, collagen I, and fibronectin (Khan et al., 2015). Another class I HDACi, MS-275, also exerted antifibrotic effects in the UUO mouse model by inhibiting TGF-β1 elevation, probably because MS-275 inhibited the expression of TGF-βR1, thereby blocking the activation of phosphorylation of the downstream signal (Smad3) and reducing the deposition of ECM in the renal interstitium (Liu et al., 2013). Yang et al. (2019) found that the application of HDAC1 and HDAC2 inhibitor FK228 slowed down the fibrosis process mediated by the pro-fibrotic factor TGF-β1 in a UUO rat model. This study found that FK228 reduced the expression of ECMs such as α-SMA, collagen I, and fibronectin; this was mediated by TGF-β1. FK228 attenuated the activation and proliferation of renal interstitial fibroblasts and promoted their apoptosis; this may stem from FK228 inhibition of the expression of the cell cycle protein CyclinD1 in fibroblasts and prevention of G1 to S phase transition of fibroblasts during proliferation. FK228 also inhibits the phosphorylation activation of Smad2/3 in the classical Smad pathway and Smad-independent pathways (ERK, p38, PI3K/AKT), which attenuates renal interstitial fibrosis in the rat model. A novel pan-inhibitor of HDACs, CG200745 (CG), exerts antifibrotic effects mainly by inhibiting class I and class II HDACs in various models of renal injury. It was found that CG can attenuate the expression of TGF-β1 mRNA and the phosphorylation of Smad2/3 in UUO mice while downregulating the accumulation of ECMs such as α-SMA, collagen I, and fibronectin (Choi et al., 2018). In a mouse model of Alport Syndrome (AS), CG was found to reverse renal fibrosis by inhibiting the RAS-mediated inflammatory response and subsequent activation of TGF-β1, and by inhibiting the phosphorylation of Smad2/3/4 and activation of myofibroblasts (Suh et al., 2020). CG inhibits the activation of the TGF-β1/Smad pathway and reduces the deposition of α-SMA, fibronectin, and collagen I in the renal interstitium, exerting an anti-fibrotic effect in a hypertensive rat model. It also inhibits the expression of inflammatory cell chemokines MCP-1, ICAM-1, and VCAM-1, ultimately exerting an anti-inflammatory effect. In the presence of CG, TUNEL analysis of DNA damage showed a significant decrease in the number of positive cells, indicating that CG inhibits apoptosis in renal tubular epithelial cells (Bae et al., 2019). In a mouse model of bilateral renal ischemia-reperfusion injury (IRI), the administration of TSA or MS275 was also effective in inhibiting the development of renal fibrosis after IRI, which was closely related to the inhibition of the TGF-β1/Smad pathway by HDACi (Hyndman et al., 2019). SB939 (pracinostat) mainly inhibits class I, II, and IV HDACs and is currently in phase II clinical trials. It was found that the administration of SB939 in a rat renal mesenchymal fibroblast model (NRK-49F) can inhibit phosphorylation of ERK, p38, and PI3K/AKT; as such, SB939 exerts its antifibrotic effects by blocking TGF-β/Smad independent pathways (Kang et al., 2017).

### 4.2 Histone deacetylases inhibitors exert antifibrotic effects by upregulating bone morphogenetic protein-7

BMP-7, a member of the TGF-β superfamily, protects against the TGF-β/Smad pathway during renal fibrosis. Sulforaphane (SFN), an inhibitor of HDAC2, can reverse renal interstitial fibrosis induced by high glucose (HG/Pal) by inhibiting HDAC2 and reactivating the BMP-7-Smad1/5/8 pathway in the mouse streptozotocin (STZ)-induced diabetes model. This suggests that upregulation of HDAC2 expression in a mouse model of diabetic nephropathy inhibits BMP-7, reduces E-cadherin formation, and promotes renal tubular epithelial-to-mesenchymal transition. In contrast, in DN, the HDAC2 inhibitor SFN reversed this process, which might prevent DN-induced renal fibrosis (Kong et al., 2021).

Yoshikawa et al. (2007) found in *in vitro* cultured human proximal tubular epithelial cells (RPTEC) that the HDAC inhibitor TSA did not reverse TGF-β1-induced phosphorylation of Smad2/3, but induced upregulation of BMP-7 and inhibitory differentiation factor (id-2). TSA reverses the TGF-β1/Smad signaling-induced down-regulation of E-cadherin and collagen I up-regulation while inhibiting the conversion of RPTEC to mesenchyme. Thereby, it enhances tubular homeostasis and delays the onset and progression of renal fibrosis. A similar study demonstrated that HDAC in a UUO mouse model regulates BMP-7 transcription. The inhibition of TGF-β1-induced Collagen-1, α1 chain (COLIα1), as well as α-SMA by TSA, was significantly reduced when the BMP-7 receptor inhibitor dorsomorphin was administered, suggesting that the HDAC inhibitor TSA counteracted TGF-β1-induced renal fibrosis by upregulating the transcription of BMP-7 (Manson et al., 2014).

### 4.3 Histone deacetylases inhibitors inhibit STAT3 activation

It has been shown that TSA inhibits activation of fibroblasts in the rat renal interstitium and attenuates the progression of renal fibrosis in UUO. The mechanism may involve HDAC activation of the transcription factor STAT3, which undergoes nuclear translocation to promote the activation of fibroblasts in the renal interstitium. At the same time, STAT3 can promote the high expression of α-SMA and fibronectin, which promote the development of renal fibrosis in CKD, a process that can be alleviated by the specific STAT3 signaling pathway inhibitors AG490 or TSA (Pang et al., 2009). It has been shown that the All-trans-retinoic acid (ATRA) and HDAC inhibitors Sodium Butyrate (NaBu) can reduce the expression of pro-inflammatory and pro-fibrotic genes downstream of NF-κB by acetylating the transcription factor STAT1, which promotes STAT1 binding to NF-κB to form a complex. This ameliorated renal interstitial fibrosis in a haploid mouse model of *Npr1* (Kumar et al., 2017).

### 4.4 Histone deacetylases inhibitors modulate inflammatory cell infiltration and inflammatory response to reduce renal interstitial fibrosis

Analysis of the renal interstitial macrophage phenotype in TSA-treated UUO mice revealed that TSA reduced the number of inducible nitric oxide synthase (iNOS)-positive M1-type macrophages in renal tissue, as well as C-type lectin domain family seven member A (CLEC7A)-positive M2a-type macrophages. These two macrophage phenotypes promote peritubular inflammation and myofibroblast activation, which further exacerbate inflammation by promoting tubular epithelial apoptosis and exacerbating immunocidal responses. However, at the same time, the number of signaling lymphocytic activation molecule (SLAM)-positive M2c macrophages in the renal interstitium was increased, which should result in anti-inflammatory effects on fibrosis formation. M2c inhibits the activation of myofibroblasts and the release of pro-fibrotic factors by promoting tubular reepithelialization and neovascularization in the damaged kidney. TSA inhibits the progression of inflammation in the injured kidney through the phenotypic transformation of macrophages M1-M2c, thus inhibiting the regression of renal tissue to fibrosis during the repair process (Tseng et al., 2020).

## 5 Summary and forecast

Chronic kidney disease seriously affects renal filtration function in patients. Its most typical pathological manifestation is fibrosis. As renal fibrosis progresses, there is a gradual loss of glomeruli and a gradual creep of blood creatinine, eventually resulting in renal failure. Although patients can benefit significantly from kidney transplantation, there is a shortage of available donors and organ rejection can lead to renal failure; many patients are forced to rely on long-term dialysis treatment. These limited treatment options have a significant impact on the morbidity and prognosis of CKD patients.

Therefore, there is a great need to identify anti-fibrosis targets at the molecular level. We found that the HDAC family may contribute to the development of renal fibrosis through non-histone epigenetic regulation. In animal models of renal fibrosis, HDACs mainly promote the TGF-β1/Smad and inhibit the BMP-7/Smad pathway. As a result, myofibroblast activation, ECM deposition, and EMT are promoted. In animal models, the application of HDACi inhibits the progression of interstitial fibrosis in the mouse kidney. Chidamide, a class I HDACi, under independent development in China, has been approved by the China Food and Drug Administration (CFDA) as a treatment for relapsed or refractory peripheral T cell lymphoma (Shi et al., 2017). We may speculate whether the application of such HDACis could similarly slow the progression of renal fibrosis in clinical patients. Such hypotheses need to be addressed by evidence from future clinical trials. However, clinical data show that the administration of HDACi is often accompanied by complications such as nausea and vomiting due to cytotoxicity (Minucci and Pelicci, 2006; Marks and Breslow, 2007). Cardiac arrhythmias, immune reactions, prolonged QT intervals, myelosuppression/thrombocytopenia, and even neurological damage and teratogenicity are known complications of HDACis (Campas-Moya, 2009; Ornoy, 2009). As such, there is an urgent need for clinical trials to identify the appropriate dose of antifibrotic treatment while screening for HDACis with high specificity and low side effects.
